# Semi-automated literature mining to identify putative biomarkers of disease from multiple biofluids

**DOI:** 10.1186/2043-9113-4-13

**Published:** 2014-10-23

**Authors:** Rick Jordan, Shyam Visweswaran, Vanathi Gopalakrishnan

**Affiliations:** 1 Department of Biomedical Informatics, University of Pittsburgh, Pittsburgh, PA, USA; 2 Intelligent Systems Program, University of Pittsburgh, Pittsburgh, PA, USA; 3 Department of Computational & Systems Biology, University of Pittsburgh, Pittsburgh, PA, USA

**Keywords:** Literature mining, Text mining, Lung cancer, Breast cancer, Biomarker, Biofluid

## Abstract

**Background:**

Computational methods for mining of biomedical literature can be useful in augmenting manual searches of the literature using keywords for disease-specific biomarker discovery from biofluids. In this work, we develop and apply a semi-automated literature mining method to mine abstracts obtained from PubMed to discover putative biomarkers of breast and lung cancers in specific biofluids.

**Methodology:**

A positive set of abstracts was defined by the terms ‘breast cancer’ and ‘lung cancer’ in conjunction with 14 separate ‘biofluids’ (bile, blood, breastmilk, cerebrospinal fluid, mucus, plasma, saliva, semen, serum, synovial fluid, stool, sweat, tears, and urine), while a negative set of abstracts was defined by the terms ‘(biofluid) NOT breast cancer’ or ‘(biofluid) NOT lung cancer.’ More than 5.3 million total abstracts were obtained from PubMed and examined for biomarker-disease-biofluid associations (34,296 positive and 2,653,396 negative for breast cancer; 28,355 positive and 2,595,034 negative for lung cancer). Biological entities such as genes and proteins were tagged using ABNER, and processed using Python scripts to produce a list of putative biomarkers. Z-scores were calculated, ranked, and used to determine significance of putative biomarkers found. Manual verification of relevant abstracts was performed to assess our method’s performance.

**Results:**

Biofluid-specific markers were identified from the literature, assigned relevance scores based on frequency of occurrence, and validated using known biomarker lists and/or databases for lung and breast cancer [NCBI’s On-line Mendelian Inheritance in Man (OMIM), Cancer Gene annotation server for cancer genomics (CAGE), NCBI’s Genes & Disease, NCI’s Early Detection Research Network (EDRN), and others]. The specificity of each marker for a given biofluid was calculated, and the performance of our semi-automated literature mining method assessed for breast and lung cancer.

**Conclusions:**

We developed a semi-automated process for determining a list of putative biomarkers for breast and lung cancer. New knowledge is presented in the form of biomarker lists; ranked, newly discovered biomarker-disease-biofluid relationships; and biomarker specificity across biofluids.

## Background

The amount of scientific information has become overwhelmingly abundant, providing querying difficulties for scientists and physicians. While many data mining and literature mining methods have been described [1-11], new and innovative methods are highly desired. Articles have been written about drawing implicit connections from separate literatures [12-15], and many unidentified connections exist within publicly available material. Identifying putative disease biomarkers may lead to new connections between biofluids and diseases being discovered.

It is known that false positive elimination from text mining findings can be aided by the use of negative abstract sets, which are abstracts that are specifically not about the entity or relationship of interest. It is also important to examine all abstracts, both positive and negative, so that the results are comprehensive and so statistical significance measures can be accurately calculated. However, it does not seem that negative abstract sets are discussed in detail.

A literature search identified several biomedical text mining papers describing the use of a negative set of abstracts [2,16-19]. Implementations of negative sets of abstracts seem to be described far less than would be expected. Adamic *et al.*[2] presented a statistical approach for finding gene-disease relations. The authors described a frequency of occurrence count and an expected number of relevant abstracts vs. a random set. Gene pairs and gene symbol disambiguation results were compared to a human edited breast cancer gene database.

Al-Mubaid, *et al.*’s method [16] for discovering protein-to-disease associations from MEDLINE abstracts employed a protein and disease name dictionary and “positive” and “negative” sets of abstracts. The positive set consisted of abstracts relevant to a given disease, as determined by a PubMed keyword search; the negative set contained a random set of abstracts that did not mention the disease. The method identified proteins that were relevant to the disease by comparing the frequency distributions of protein names in the positive set and the overall set, which was the union of the positive and negative sets, and selected those proteins for which the distributions were significantly different statistically.

Andrade [17] was interested in annotating biological function of protein sequences. In this article, the ‘treatment of text with statistical methods’ was discussed. Their approach estimated the word significance from a given set of protein family abstracts by comparing each word’s abundance and distribution in a background set of varying protein family abstracts.

Younesi, *et al.*[18,19] divided the biomarker terminology into six concept classes (clinical management; diagnostics; prognosis; statistics; evidence; and antecedent). By including this extra level of restriction, the authors were able to significantly reduce the number of retrieved relevant documents. Frequency and entropy ranking methods were used for acquired genelists, with frequency ranking performing better overall, with their method.

Individual biofluids have been characterized; [20-25] however, we have found only one comprehensive comparison of more than a few biofluids. Alterovitz *et al.*[26] compared 10 biofluid proteomes to 16 tissue proteomes to determine tissue function, and tissue-specific candidate biomarkers that could be found in a given biofluid. Gene Ontology (GO); [27,28]http://www.geneontology.org/, was used for functionality mapping, NCBI’s Online Mendelian Inheritance in Man (OMIM); [29]http://www.ncbi.nlm.nih.gov/omim/, for disease mapping, the Pharmacogenomics Knowledge Base (PharmGKB); [30]https://www.pharmgkb.org/, for drug mapping, and a relative entropy measure was the scoring method of choice. PubMed co-citation frequencies were used to determine the overall quality of the candidate biomarkers.

Comparisons such as those described above have the potential to reveal critical knowledge as to which biomarkers for a disease may be detected in a given biofluid. As some biofluids are more easily obtainable than others, elimination of invasive sampling procedures is highly desirable. However, details describing which potential biomarkers can be obtained in given biofluids are not clearly defined.

In this paper, we developed a semi-automated process for determining a list of putative biomarkers for breast and lung cancers, with a putative biomarker being described as a ‘gene’ or ‘protein’. 5.3 million PubMed abstracts were analysed for biomarker-disease associations (34,296 positive and 2,653,396 negative for breast cancer; 28,355 positive and 2,595,034 negative for lung cancer). The abstract sets were further stratified among 14 biofluids. New knowledge is provided in the form of known disease biomarker lists, ranked newly discovered biomarker-disease-biofluid relationships, and biomarker specificity across biofluids. On average, (see Additional file 1) we expect true positive rates for new discoveries to be 87.5% for breast cancer, and 71.59% for lung cancer. These biomarker-disease association and accompanying z-scores will be used as informative prior values in future disease modeling activities.

## Methodology

### Automation

**Figure 1:**
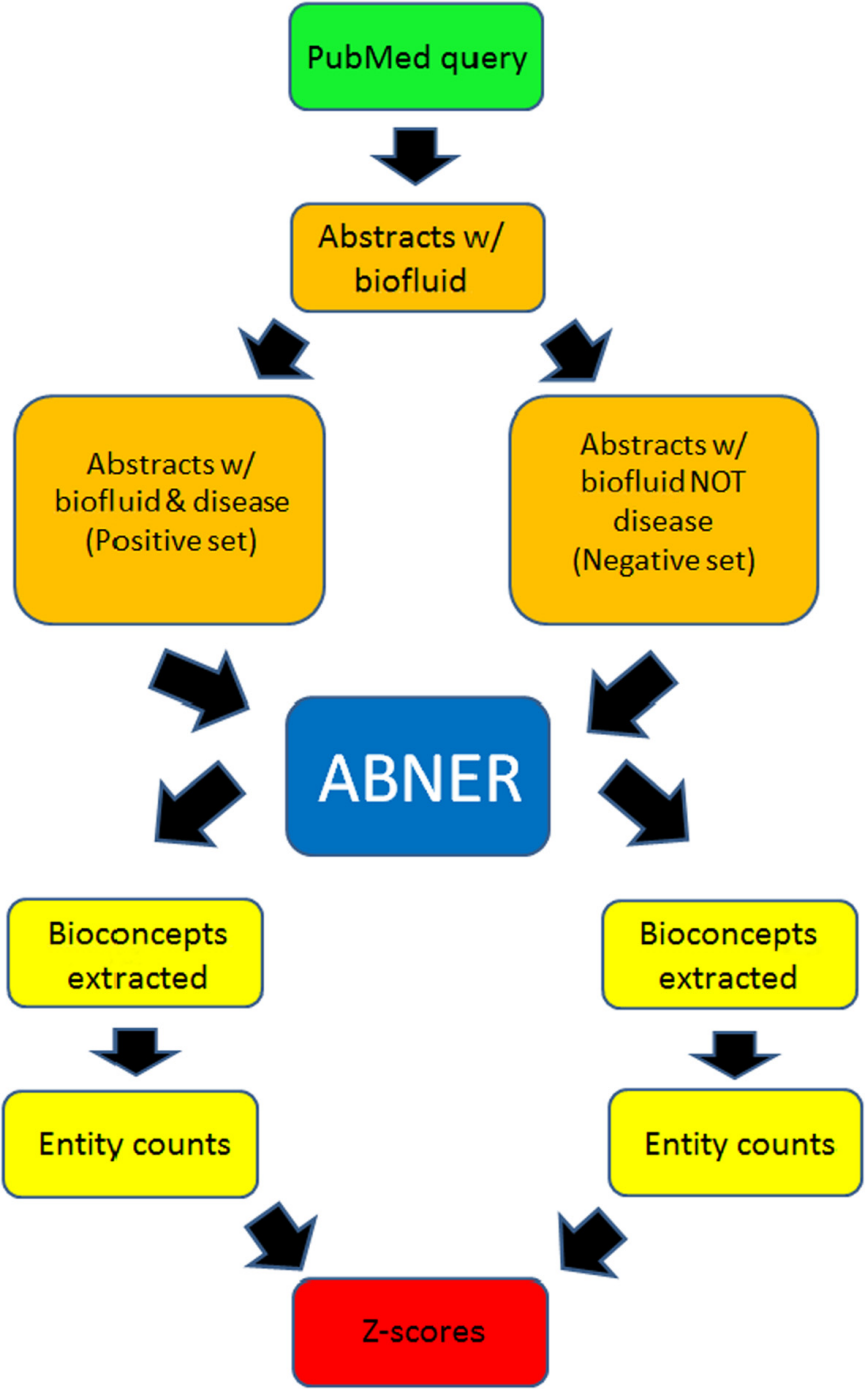
**Semi-automated flowchart of the information retrieval process.** Python scripts were written to process text files. ABNER was used for tagging biological entities, and the z-score calculation was performed using Microsoft Excel.

Python scripts were developed to reduce the amount of manual effort needed to achieve final scores for each potential biofluid biomarker, and to eliminate manual errors. Figure 1 shows a flowchart that summarizes the experimental methodology used.

### Information retrieval

For retrieving abstracts related to breast and lung cancer, a PubMed query was performed using the following limits: Abstracts, English, and Human. Query results for diseases-biofluid can be found in Table 1 (see Additional file 2 for Biofluid synonyms used). An abstract consists of journal entry information, title, authors, affiliations, text, copyright information, and PubMed ID. The following sets of abstracts were obtained using the selected criteria from the positive and/or negative queries (defined below).

**Table 1:** Size of the abstract sets returned from queries of breast and lung cancer

**Breast cancer**			**Lung cancer**		
**Biofluid**	**Positives**	**Negatives**	**Biofluid**	**Positives**	**Negatives**
Bile	360	40,250	Bile	328	40,290
Blood	18,939	1,540,721	Blood	15,710	1,522,046
Breastmilk	1,047	17,874	Breastmilk	99	18,834
CSF	252	42,711	CSF	298	42,676
Mucus	116	25,122	Mucus	1,445	23,801
Plasma	4,327	342,415	Plasma	3,227	343,678
Saliva	149	22,694	Saliva	86	22,770
Semen	40	12,956	Semen	9	12,989
Serum	7,410	415,218	Serum	6,029	412,897
SF	18	7,699	SF	18	7,671
Stool	123	37,574	Stool	90	37,619
Sweat	321	11,079	Sweat	88	11,673
Tears	40	11,651	Tears	10	11,673
Urine	1,154	125,462	Urine	918	86,776
Total	34,296	2,653,396	Total	28,355	2,595,034

CSF = cerebrospinal fluid; SF = synovial fluid.

• ***Positive Abstract Sets***

• A positive abstract set is defined as the set of abstracts obtained by using the following combination of keywords, ‘breast cancer AND (biofluid)’, e.g. breast cancer AND plasma, or ‘lung cancer AND (biofluid)’. From this point forward, all positive abstract sets will be called “positive sets” for brevity. Positive set queries were performed on 4-29-2013 for breast cancer and 5-2-2013 for lung cancer. The underlying assumption being made is that any possible biomarker mentioned in these abstract sets is related to both the disease and the biofluid. Queries were returned from PubMed as large text files, and Python scripts were implemented to process the files.

• ***Negative Abstract Sets***

• We define a negative abstract set as a set of abstracts returned using the keywords ‘(biofluid) NOT breast cancer’ or ‘(biofluid) NOT lung cancer’. From this point forward, all negative abstract sets will be called “negative sets” for the entirety of this article. Negative set queries were performed on 4-29-2013 for breast cancer and 5-2-2013 for lung cancer. Queries were returned from PubMed as large text files, and Python scripts were implemented to process the files.

### Filtering information

Python scripts were developed to remove unwanted punctuation and other unwanted information from the abstracts.

### Named entity recognition

ABNER [31] (A Biomedical Named Entity Recognizer; http://pages.cs.wisc.edu/~bsettles/abner/) v1.5 was used to tag mentions of proteins, DNA, RNA, cell lines, and cell types in the positive and negative sets. Version 1.5 trains on the NLBPA and BioCreative corpora. Reported performance measures for ABNER are in the range of 65.9-77.8 for protein recall and 68.1-74.5 for protein precision. Our method utilizes entities tagged as “Protein”, “DNA”, and “RNA”. A batch tagging process is available and proved to be extremely useful.

### Entity extraction

Python scripts were developed to produce a list of tagged entities from the ABNER results file (.sgml), remove unwanted characters, tags, tagged entries, and duplicate putative biomarkers from the list, and to tally the final count of each biological entity found. PubMed identifiers were retained for tracking and manual verification purposes.

### Dictionary

A file named Protein Nomenclature was downloaded from the Human Protein Reference Database Copyright^©^ 2002-09, Johns Hopkins University and The Institute of Bioinformatics (Additional file 3), to use as a dictionary file. The file contains 19,327 unique IDs. The format consists of the HPRD id, gene symbol, RefSeq id, and aliases (separated by semi-colons). The gene symbol will be used to create a consensus name for all other aliases found. The entities were mapped via another Python script.

### Scoring

Counts were performed at the abstract level, where a mention of a given biomarker was assigned a count of 1, regardless of the frequency of mentions within the abstract.

Each z-score corresponds to a point in a normal distribution and can be associated to its deviation from the mean. Z-scores were computed as follows:

Briefly, from Al-Mubaid [16], *S*_*1*_ is the positive set of abstracts (i.e. disease/biofluid), *S*_*1*_ = {*A*_*1*_, *A*_*2*_, …, *An*}. *A* is a given abstract, *S*_*p*_ is the set of proteins (markers) mentioned in the dictionary found in the positive set *S*_*1*_, *S*_*p*_ = {*P*_*1*_, *P*_*2*_, …, *P*_*m*_}. *S*_*2*_ is the negative set of abstracts.

For each protein (marker) *P*_*i*_ in *S*_*p*_, compute the document frequency (df) of *P*_*i*_ in both sets *S*_*1*_ and *S*_*2*_ as:

$$\begin{array}{c} df1\left(P_{i}\right)=\text{number}\;\text{of}\;S_{1}\;\text{documents}\;\text{in}\;\text{which}\;P_{i}\\ \;\text{is}\;\text{mentioned}, \end{array}$$

$$\begin{array}{c} df2\left(P_{i}\right)=\text{number}\;\text{of}\;S_{2}\;\text{documents}\;\text{in}\;\text{which}\;P_{i}\\ \;\text{is}\;\text{mentioned}, \end{array}$$

$$dft\left(P_{i}\right)=df1\left(P_{i}\right)+df2\left(P_{i}\right)\text{.}$$

For each protein in the set Sp compute an expectation (ex) value and an evidence (ev) value as:

$$\text{ex}\left(P_{i}\right)=\left[dft\left(P_{i}\right)/\left|S_{1}+S_{2}\right|\right]*\left|S_{1}\right|,\;\text{and}$$

$$ev\left(P_{i}\right)=df1\left(P_{i}\right)$$

Ex measures expected number of mentions of *P*_*i*_ in the abstracts in set *S*_*1*_; ev measures actual number of *S*_*1*_ abstracts that *P*_*i*_ has appeared in. The larger the difference in observed and expected document frequencies, ev(*P*_*i*_) – ex(*P*_*i*_), the more likely that *P*_*i*_ and the disease are significantly associated.

The difference is normalized by:

$$f\left(P_{i}\right)=ev\left(P_{i}\right)-\text{ex}\left(P_{i}\right)/dft\left(P_{i}\right)\text{.}$$

And the z-score is calculated by:

$$Z\left(P_{i}\right)=\left[f\left(P_{i}\right)-\text{mean}\left(f\right)\right]/SD\left(f\right)$$

where mean(f) is the mean of all f values of all proteins of *S*_*p*_ and SD(f) is the standard deviation of the f values.

**Figure 2:**
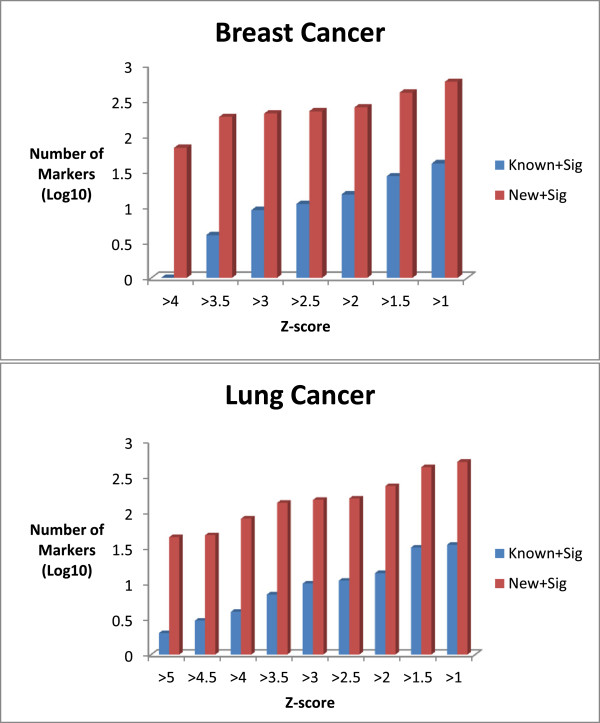
**Number of markers identified across the range of possible Z-scores.** Decreasing the Z-score threshold allows for more significant markers to be identified.

A threshold value of 1.0 was established as a significance cut-off (see Figure 2). These z-score values will be used as informative prior values in future modeling efforts (Additional file 4 and Additional file 5).

### Verification of relationships

One possible method of verification is to remove from the abstract pool, ‘verification documents’ (ones specifically pertaining to a disease-protein relationship), and use them for subsequent verification [16]. Our method allows these abstracts to remain in the pool, and verification is performed by comparing our results to a combined disease biomarker list (Additional file 6: Table S1 & Additional file 7: Table S2). The list was created using the following sources: OMIM [29] (O in table); http://www.ncbi.nlm.nih.gov/omim/), a cancer gene annotation system for cancer genomics [32] (CAGE(C); http://mgrc.kribb.re.kr/cage/pageHome.php?m=hm), NCBI’s Genes & Disease [33] ((G); http://www.ncbi.nlm.nih.gov/books/NBK22183/), NCI’s Early Detection Research Network [34] (EDRN (E); http://edrn.nci.nih.gov/), an expert provided list (X) of validated cancer markers [35], and a recently released breast cancer paper [36] (P). Markers that are present in at least one of these lists, as well as in our dictionary were considered verified. The list for breast cancer was compiled using OMIM, CAGE, Genes & Disease, the expert provided list, and the previously mentioned paper. The lung cancer list was compiled from OMIM, CAGE, EDRN, and the expert provided list.

### True positive rate determination

Negative abstracts were utilized to initially eliminate some false positives. However, it is more likely than not, that this process alone will not completely eliminate all false positives.

In processing the abstracts, it was apparent that eventually manual examination of abstracts would be required for result verification. The abstract PubMed identifier of every possible instance of every biomarker mention accompanied each biomarker, allowing for manual tracking and further verification of our results. Relevant abstracts were investigated further. Three criteria were used for a pass/fail outcome. Abstracts were examined for mentions of biomarker, disease, and biofluid. All three criteria were required to be acceptable, and synonyms and/or root words were deemed adequate (e.g. biliary instead of bile).

## Results

### Positive and negative sets

Table 1 describes the number of relevant abstracts obtained from the PubMed searches. Fourteen biofluids were evaluated. From this table, blood, plasma, and serum returned the most positive and negative abstracts from both breast and lung cancer queries. Over five million total abstracts were examined.

### Known markers per biofluid

Our known marker lists are combinations of several ‘biomarker lists’ obtained from well-known databases. The known breast cancer marker list contains 211 gene symbols that mapped to our dictionary (Additional file 6: Table S1; 159 found in this exercise), and the known lung cancer marker list has 209 markers that mapped to our dictionary (Additional file 7: Table S2; 145 found in this exercise). Known marker results presented in Table 2 were obtained by identifying putative biomarkers with a z-score exceeding the significance threshold (>1.0), and confirming the gene symbol in our known disease biomarker list. Table 2 also summarizes the biofluids that produced markers with significant z-scores and/or the number of known markers found for breast and lung cancer.

**Table 2:** Number of markers identified for each disease-biofluid combination

**Breast Cancer**	**Total number of markers found**	**Known markers found (211 possible)**	**Markers producing a significant z-score (>1.0)**	**Known markers with a significant z-score**	**New markers with a significant z-score**	**% new discoveries**
Bile	200	26	58	7	51	87.93
Blood	2084	150	196	9	187	95.41
Breastmilk						
CSF	116	8	18	0	18	100.00
Mucus	63	13	8	3	5	62.50
Plasma	1002	88	100	5	95	95.00
Saliva	73	9	10	2	8	80.00
Semen	35	3	6	0	6	100
Serum	1327	106	145	6	139	95.86
SF	21	0	4	0	4	100.00
Stool	68	8	7	3	4	57.14
Sweat	123	15	28	3	25	89.29
Tears	26	2	3	0	3	100.00
Urine	310	32	38	3	35	92.11
**Lung Cancer**	**Total number of markers found**	**Known markers found (211 possible)**	**Markers producing a significant z-score (>1.0)**	**Known markers with a significant z-score**	**New markers with a significant z-score**	**% new discoveries**
Bile	167	17	25	1	24	96.00
Blood	1863	141	152	7	145	95.39
Breastmilk	77	15	11	2	9	81.82
CSF	106	7	11	1	10	90.91
Mucus	276	27	73	10	63	86.30
Plasma	843	75	65	4	61	93.85
Saliva	53	3	7	1	6	85.71
Semen	11	2	0	0	0	0
Serum	1109	100	103	3	100	97.09
SF	13	2	3	0	3	100.00
Stool	45	2	5	0	5	100.00
Sweat	44	5	4	0	4	100.00
Tears	12	0	1	0	1	100.00
Urine	256	30	56	6	50	89.29

Known markers were determined by identification of the given gene symbol in our known biomarker lists (Additional file 6: Table S1 or Additional file 7: Table S2). Significant markers had a z-score >1.0.

### Z- score threshold optimization

We chose an appropriate threshold for z-score based on empirical findings. As shown in Figure 2 which is a plot of the number of known markers and new markers (log_10_) based on the z-score threshold which was varied between 1 and 4 in increments of 0.5. Based on this we chose a non-stringent z-score threshold of 1.0 which allows us to identify the maximum number of known and new markers.

### Comparison of identification of potential biomarkers by disease-biofluid

Table 2 shows the breakdown of the number of markers found by our method. In most biofluids, the number found in breast cancer outnumbers the number found in lung cancer, with the exceptions being breastmilk (removed from our breast cancer examination due to both positive and negative search terms containing the root ‘breast’) and mucus (greater association with respiratory system).

### Known markers found significant vs. non-significant

While the truth is unknown as to the members of the comprehensive pool of breast or lung cancer biomarkers, and thus a true positive value cannot be obtained, estimates can be made. Although these numbers are not shown, one can easily calculate the percentage of known markers identified as significant vs. not-significant using the counts from Table 2.

For breast cancer, percentages range from 5% in plasma and serum to 37.5% in stool (for biofluids with known-significant markers; non-zero). In lung cancer the range is from 3% in serum to 37% in mucus.

### Newly discovered markers found significant vs. non-significant

The percentage of newly discovered markers (markers not found in known marker list) that were found to be significant vs. the percentage that were identified but not found to be significant was calculated.

For breast cancer, percentages range from 6.67% in stool to 29.3% in bile (for biofluids with known-significant markers; non-zero). In lung cancer the range is from 7.9% in plasma to 27.2% in synovial fluid.

### Potential marker biofluid specificity

Biomarker commonality and specificity was sought across biofluids. This was a significant finding in that we have not seen many potential biomarker comparisons across more than a few biofluids. Additional file 8: Table S3 shows the known + significant biomarkers within biofluids for breast and lung cancer.

A total of 21 known + significant markers were identified for breast cancer. Nine biofluids produced known ID’s with significant scores. A breakdown of this list shows that 14 are only identified in combination with one biofluid, 3 with two biofluids, 1 with 3 biofluids (ERBB2; mentioned blood, plasma, and serum), 1 with 4 biofluids (NCOA3; mentioned in bile, blood, plasma, and serum), 1 with 6 biofluids (BRCA2; mentioned in bile, blood, mucus, saliva, serum, and sweat), and 1 with 7 biofluids (BRCA1; mentioned in blood, mucus, plasma, saliva, serum, sweat, and urine abstracts).

A total of 26 known + significant putative markers were identified for lung cancer. Eight biofluids produced known ID’s with significant scores. A breakdown of this list shows that 21 are only mentioned in combination with one biofluid, 3 with two biofluids, 1 with 3 biofluids (EML4; mentioned in blood, mucus, and serum), and 1 with 4 biofluids (KRAS; mentioned in blood, breastmilk, mucus, and serum).

### Manual verification of findings

A manual check of relevant abstracts was performed to ensure the reliability of our results. Each relevant PubMed abstract was manually examined to verify the biomarker mentioned. The results of this manual verification can be seen in Additional file 1: Table S4. Four known biomarkers (CHEK2 in both plasma and urine, CDKN1B, PCNA, and THBS1) were identified as false positives (red) in our breast cancer list, and seven (KRAS, GDNF in both breastmilk and plasma, MYCL1 in both blood and serum, CD40LG, CGA, CTAG1A, ERCC6, and HRAS) in our lung cancer list. KRAS is interesting in that it produced a false positive in association with breastmilk, but had verified positive findings in associations with blood, mucus, and serum.

### True positive rate estimation of new discoveries

Manual verification allowed us to calculate the true positive rates across the biofluids-diseases. The results found in Additional file 1: Table S4 show an average error rate for breast cancer of 12.5%, and an average lung cancer error rate of 29.41%. From these calculations, one can conclude that 87.5% of the breast cancer new discoveries would be true positives, and 70.59% of the lung cancer new discoveries would be true positives.

## Discussion

We have presented a method to determine the possibility of relatedness between potential biomarkers in biofluids and disease (breast and lung cancers), using positive and negative sets of abstracts and a z-score.

Error exists in ABNER’s [31] tagging, our dictionary consensus, and possibly anywhere manual processing of the data occurs. Negation was not addressed at this time.

A potential dictionary problem was identified in that some members of a protein family had a generic alias in common. This led to results such as ceacam5 and ceacam8 both being identified for the CEA alias. Adding another unique ID such as “ceacam_family” to account for this double counting was considered, however it was decided to let the counts stand, as there may be double counting elsewhere in the dictionary of which we are unaware.

In some situations a potential biomarker may need to only be mentioned in one negative set abstract to exhibit non-significance by our method. As disease-specific potential markers are sought, common biomarkers implicated in several diseases may not reach a significant score by our method because of their mention in abstracts describing other diseases including other types of cancer.

A requirement for potential biomarkers to appear in different abstracts was not applied. Several biomarker mentions may come from the same abstract. Similarly, there was not a requirement for different biofluids to appear in different abstracts. One biomarker discussed in association with more than one biofluid may appear in the list for each biofluid.

The number of known cancer biomarkers found but deemed not significant was reported. The results may be due to the way the negative search space was defined. It is possible that abstracts of other cancers or diseases exist in our negative set, and thus any biomarker mentioned in association with any other disease would negate our positive findings for breast and/or lung cancer.

Databases used for verification are probably far from being complete, which could be why our list of known + significant biomarkers is smaller than expected. Another explanation could be that certain markers just may not be found in a given biofluid. We will work to improve our verification methods over time.

Lastly, only abstracts were examined in this work. Obviously, full text examination would produce more findings as well as more confidence in the findings, but access to full text remains a limiting factor for all text-mining researchers.

## Conclusions

We have presented a method that utilizes literature mining to create a list of documented putative biomarker-biofluid relationships for breast and lung cancer. Over 5 million abstracts were analyzed for biomarker-disease associations. These abstract sets were further stratified among 14 biofluids. Some false positives were initially eliminated by examining negative sets of abstracts and establishing a threshold z-score. New knowledge pertaining to breast and lung cancer is presented in the forms of known disease biomarker lists; ranked, newly discovered biomarker-disease-biofluid relationships; and biomarker specificity across biofluids. The relationships obtained from literature mining were verified by comparison to well-known published databases. Manual examination of abstracts allowed for known relationship verification and true positive rate calculations. On average, we can expect an 87.5% true positive rate for our breast cancer new discoveries, and a 71.59% true positive rate for our lung cancer new discoveries.

Future work in this area will include further automation of our semi-automated process, applying our method to other diseases, assembling a disease database to make our z-score findings available to others, as well as converting our z-score values into prior probabilities for use as informative priors in Bayesian disease modeling.

## Competing interests

The authors declare that they have no competing interests.

## Authors’ contributions

RJ wrote the Python scripts, downloaded abstracts, performed analysis, created figures and tables. VG conceived of the study, participated in its design and coordination. SV provided methodology and participated in study design. All authors participated in drafting the manuscript as well as reading and approving the final manuscript.

## Supplementary Material

Additional file 1: Table S4 Manually verified biomarker table. Biomarker specific abstracts were manually examined for accuracy. Abstracts were examined for mentions of biofluid, disease, and biomarker. Lack of any one term resulted in a ‘false positive’ result. Click here for file

Additional file 2 SupplementaryBiofluidTable. Click here for file

Additional file 3 SupplementaryProteinlist. Click here for file

Additional file 4 SupplementaryBCResults. Click here for file

Additional file 5 SupplementaryLCResults. Click here for file

Additional file 6: Table S1 List of breast cancer identifiers. Click here for file

Additional file 7: Table S2 List of lung cancer identifiers. Click here for file

Additional file 8: Table S3 **Identification of the significant validated potential markers found to be in common to several biofluids or biofluid specific for breast and lung cancer.** Biomarkers highlighted in yellow are either breast cancer markers found in the list of validated lung cancer biomarkers (Additional file 7: Table S2), or lung cancer markers found in the list of validated breast cancer biomarkers (Additional file 6: Table S1). It is doubtful that these markers are disease specific. CDH1 is the only found biomarker in both cancer lists. Click here for file
