# Supplementary figures and images for: Semi-automated literature mining to identify putative biomarkers of disease from multiple biofluids
Source: J Clin Bioinforma. 2014 Oct 23;4:13. doi: 10.1186/2043-9113-4-13 (PMC4215335; doi:10.1186/2043-9113-4-13)

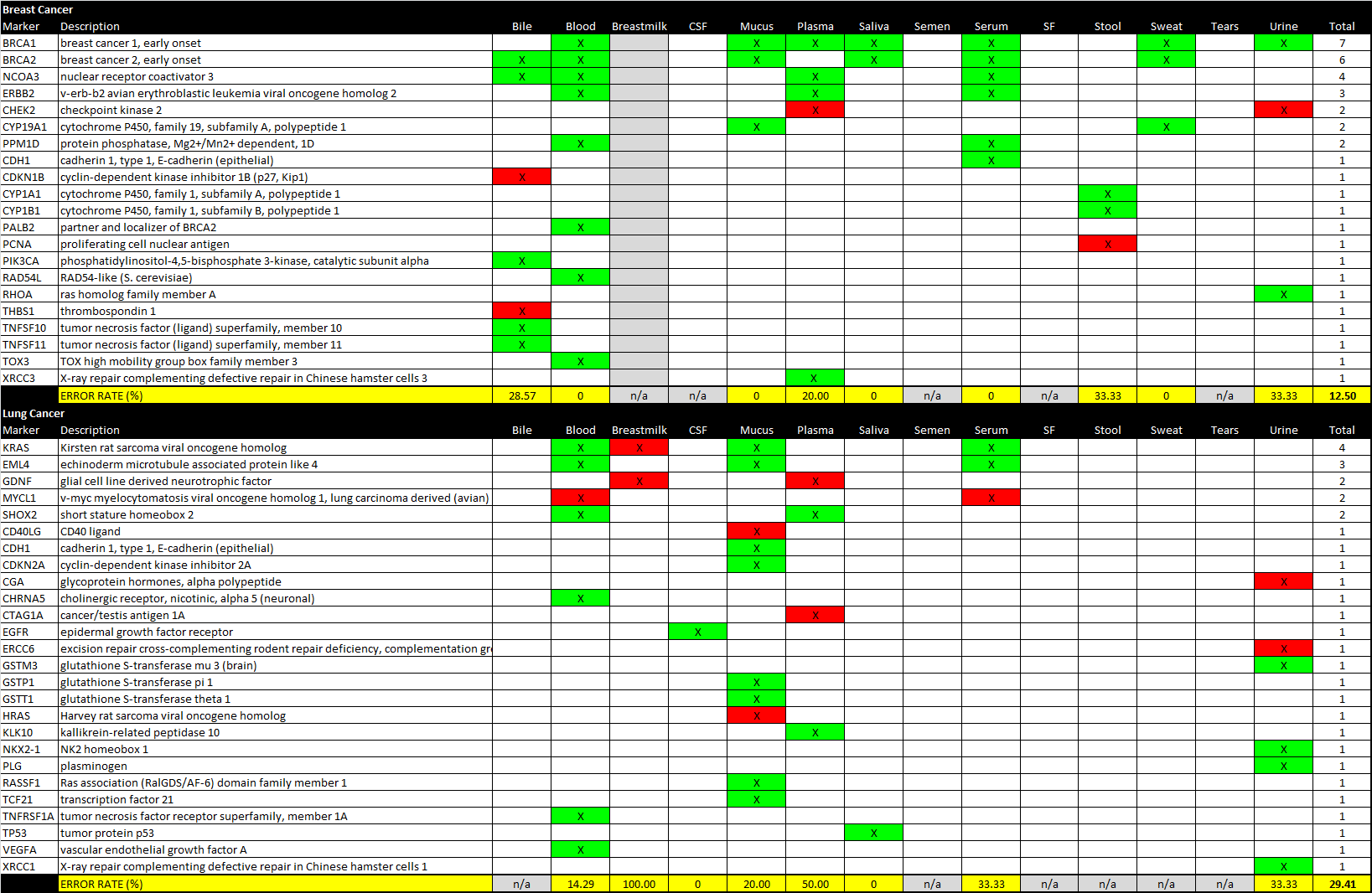

Supplement: Additional file 1: Table S4 — Manually verified biomarker table. Biomarker specific abstracts were manually examined for accuracy. Abstracts were examined for mentions of biofluid, disease, and biomarker. Lack of any one term resulted in a ‘false positive’ result. [file 2043-9113-4-13-S1.docx]

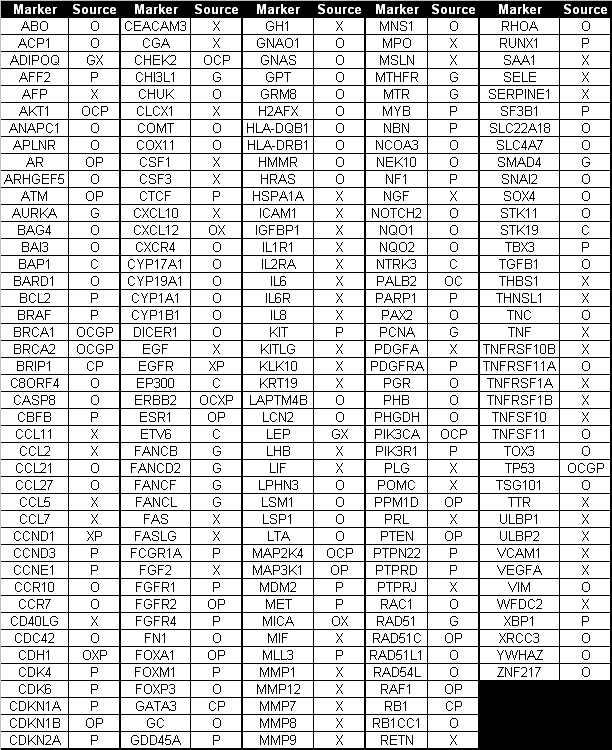

Supplement: Additional file 6: Table S1 — List of breast cancer identifiers. [file 2043-9113-4-13-S6.docx]

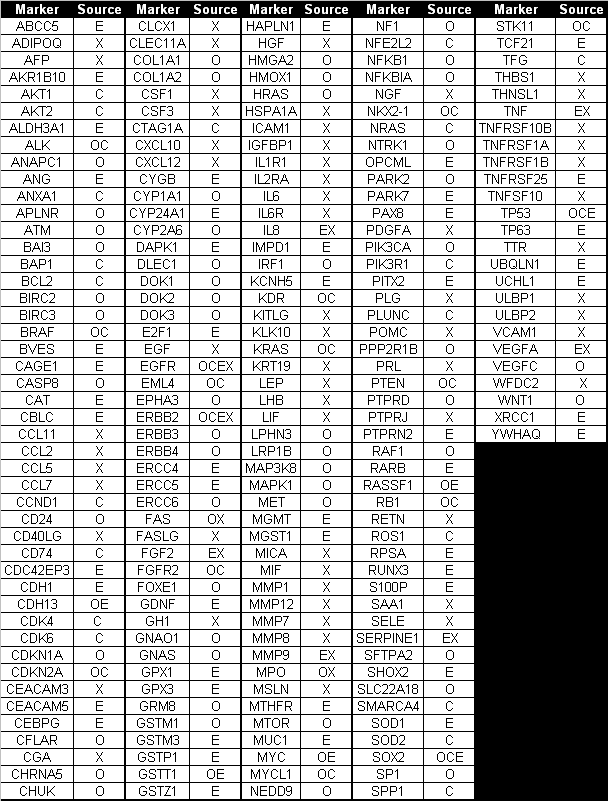

Supplement: Additional file 7: Table S2 — List of lung cancer identifiers. [file 2043-9113-4-13-S7.docx]

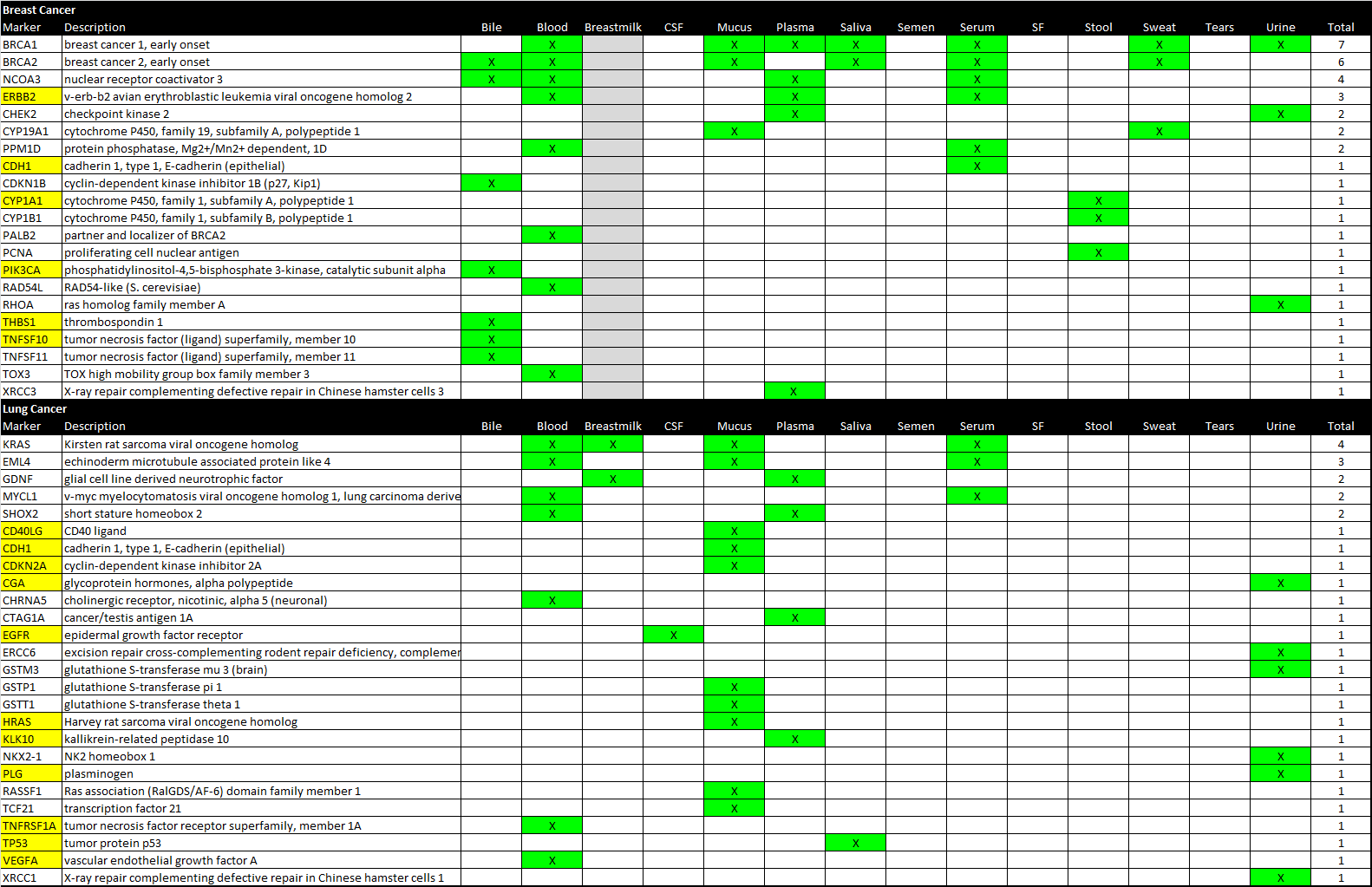

Supplement: Additional file 8: Table S3 — Identification of the significant validated potential markers found to be in common to several biofluids or biofluid specific for breast and lung cancer. Biomarkers highlighted in yellow are either breast cancer markers found in the list of validated lung cancer biomarkers (Additional file 7: Table S2), or lung cancer markers found in the list of validated breast cancer biomarkers (Additional file 6: Table S1). It is doubtful that these markers are disease specific. CDH1 is the only found biomarker in both cancer lists. [file 2043-9113-4-13-S8.docx]
